# Dual Virucidal and Direct Antibacterial Activities of a Commercial Green Tea Extract Preparation and EGCG: Potential Applications for Oral and Upper Respiratory Health

**DOI:** 10.3390/antibiotics15101007

**Published:** 2026-10-10

**Authors:** Kwang Mi Kim, Su Hyeon An, Ju Won Kim, Ye Bin Park, Seonjeong Kim, Sujeong Kim, Young Ho Byun, Sera Moon, Yo Han Jang

**Affiliations:** 1 Vaccine Biotechnology Major, Gyeongkuk National University, Andong 36729, Republic of Korea; km030421@naver.com (K.M.K.); suhyeon0628@naver.com (S.H.A.); wn1373@naver.com (J.W.K.); 027408@naver.com (Y.B.P.); 2 Research Institute, GreenVax Inc., Incheon 21983, Republic of Korea; kksj9173@greenvax.co.kr (S.K.); tndlwjd@greenvax.co.kr (S.K.); greentearsr2022@greenvax.co.kr (Y.H.B.); serabiotech@greenvax.co.kr (S.M.)

**Keywords:** green tea extract, EGCG, influenza virus, virucidal activity, antibacterial activity, oral bacteria

## Abstract

**Background/Objectives**: Green tea-derived catechins, including epigallocatechin gallate (EGCG), possess diverse antimicrobial properties. This study investigated the dual antiviral and antibacterial activities of a commercially available green tea extract (cGTE) preparation and EGCG against influenza viruses and representative oral bacteria to evaluate their potential for oral and upper respiratory applications. **Methods**: The antiviral activities of the cGTE preparation and EGCG against influenza A virus strains H1N1 (A/Puerto Rico/8/1934) and H5N2 (mouse-adapted influenza A/aquatic bird/Korea/w81/2005) were evaluated by assessing virucidal activity, time-of-addition experiments, and inhibition of hemagglutinin (HA) and neuraminidase (NA) activities. Antibacterial activity was assessed following direct exposure of Gram-positive oral streptococci (*Streptococcus mutans*, *S. salivarius*, *S. mitis*, *S. sanguinis*, and *S. sobrinus*), *Staphylococcus aureus*, and Gram-negative *Escherichia coli* strains to the cGTE preparation or EGCG. **Results**: Both the cGTE preparation and EGCG exhibited concentration- and time-dependent virucidal activities against A/H1N1 and A/H5N2 viruses. Direct treatment of the viruses generally produced greater antiviral effects than cell pre- or post-treatment. Both treatments also inhibited HA and NA activities, suggesting that interference with viral surface-associated functions may contribute to their antiviral effects. In addition, the cGTE preparation and EGCG reduced bacterial viability following direct exposure, with EGCG showing strong direct antibacterial activity against oral *Streptococcus* species. **Conclusions**: The cGTE preparation and EGCG exhibited dual virucidal and direct antibacterial activities against influenza viruses and a range of oral bacteria. These findings support their potential as multifunctional antimicrobial agents for locally applied formulations targeting the oral and upper respiratory environments.

## 1. Introduction

Influenza viruses are major respiratory pathogens that cause seasonal epidemics and continue to impose a substantial global burden of disease [1]. Vaccination remains the primary strategy for influenza prevention, while antiviral drugs are used for the treatment and control of infection [2,3]. However, the continuous evolution of influenza viruses and the emergence of antiviral-resistant variants highlight the need for complementary approaches to infection control [4,5]. In addition to agents that inhibit viral replication within infected cells, substances capable of directly inactivating viral particles may be useful for locally applied antimicrobial strategies, particularly when direct contact between the active ingredient and infectious viral particles can be achieved in the oral or upper respiratory environment [6,7,8,9,10,11].

Green tea (*Camellia sinensis*) contains abundant polyphenolic compounds, particularly catechins, among which epigallocatechin gallate (EGCG) is one of the major bioactive constituents [12]. Green tea-derived catechins, including EGCG, have been reported to exhibit antiviral activity against influenza viruses through interference with multiple stages of the viral life cycle, including viral attachment, entry, and replication. In addition, catechins have been shown to affect the functions of influenza virus surface proteins, including HA and NA [13,14,15]. These findings suggest that green tea-derived compounds may exert antiviral activity through direct interactions with viral particles as well as interference with virus-associated functions. However, the antiviral effects of these compounds can vary depending on the viral strain, concentration, and treatment conditions, and further evaluation of their direct virucidal activity remains important when considering their use as locally applied antimicrobial ingredients. The oral cavity represents another environment in which direct antimicrobial activity may be beneficial. Oral streptococci, including *Streptococcus mutans*, *S. salivarius*, *S. mitis*, *S. sanguinis*, and *S. sobrinus*, are common members of the oral microbiota that contribute to the establishment and ecological dynamics of oral biofilms, with individual species playing distinct roles in oral health and disease [16]. Among these species, *S. mutans* and *S. sobrinus* are well-recognized cariogenic bacteria associated with dental caries, whereas other oral streptococci contribute to microbial colonization and maintenance of the oral microbial community [17]. Previous studies have primarily focused on the effects of green tea catechins, particularly EGCG, against *S. mutans*, demonstrating inhibitory effects on bacterial growth, viability, acid production, virulence factors, and biofilm formation [18]. Although several other oral streptococci, including *S. sobrinus*, *S. salivarius*, and *S. sanguinis*, have also been investigated, their responses to green tea-derived materials have been evaluated less extensively [19,20,21]. Therefore, comparative evaluation of multiple oral streptococcal species under a common experimental framework may provide a broader understanding of the antibacterial spectrum of green tea extract (GTE) and its major catechin EGCG. Furthermore, growth inhibition and direct bacterial killing do not necessarily represent the same antimicrobial activity. Evaluation of viable bacterial cells following direct exposure may therefore provide complementary information regarding the potential of green tea-derived materials as locally applied antimicrobial agents. GTE is a complex mixture containing EGCG and other catechins and polyphenolic compounds, whereas purified EGCG represents a major individual bioactive component of green tea [22]. Comparing GTE with EGCG may therefore help determine whether the antimicrobial activities of the extract are similarly reproduced by its major catechin. Importantly, although the antiviral and antibacterial properties of green tea-derived compounds have been individually investigated, the simultaneous evaluation of their direct virucidal and bactericidal activities against respiratory viruses and oral bacteria may provide a broader perspective on their potential as multifunctional antimicrobial ingredients.

In the present study, we investigated the direct antiviral and antibacterial activities of a commercially available GTE (cGTE) preparation and EGCG against influenza A viruses and representative oral bacteria. The virucidal activities of the cGTE preparation and EGCG were evaluated against influenza A/H1N1 and A/H5N2 viruses, together with time-of-addition experiments (TOA) and analyses of hemagglutinin (HA) and neuraminidase (NA) activities to further characterize their antiviral effects. Their antibacterial activities were evaluated against five oral *Streptococcus* species, *Staphylococcus aureus*, and representative *E. coli* strains using the agar well diffusion and bactericidal assays. Because cGTE is a chemically complex preparation containing multiple catechins and other green tea constituents, whereas EGCG is a chemically defined and quantitatively prominent catechin, their parallel evaluation provides a scientifically relevant framework for distinguishing the antimicrobial activity of the whole extract from that of a representative individual constituent. Although EGCG is widely recognized as a major bioactive component of green tea, it remains unclear whether the antimicrobial activity observed with a commercial green tea extract can be explained primarily by EGCG or whether the extract exhibits a distinct activity profile resulting from its multiple constituents. Direct comparison of cGTE and EGCG against both viruses and bacteria therefore allow their antiviral and antibacterial spectra to be examined under comparable experimental conditions and may reveal differences in activity that cannot be inferred from studies of either material alone. In this context, the present study evaluated the effects of cGTE and EGCG on viral infectivity and bacterial viability following direct exposure, with particular emphasis on their dual antiviral and antibacterial activities. This comparative approach was intended to characterize the activity profiles of the commercial extract and its representative major constituent and to provide a scientific basis for considering their potential use in locally applied formulations targeting the oral and upper respiratory environments.

## 2. Results

### 2.1. Cytotoxicity of the cGTE Preparation and EGCG in MDCK Cells

**Figure 1:**
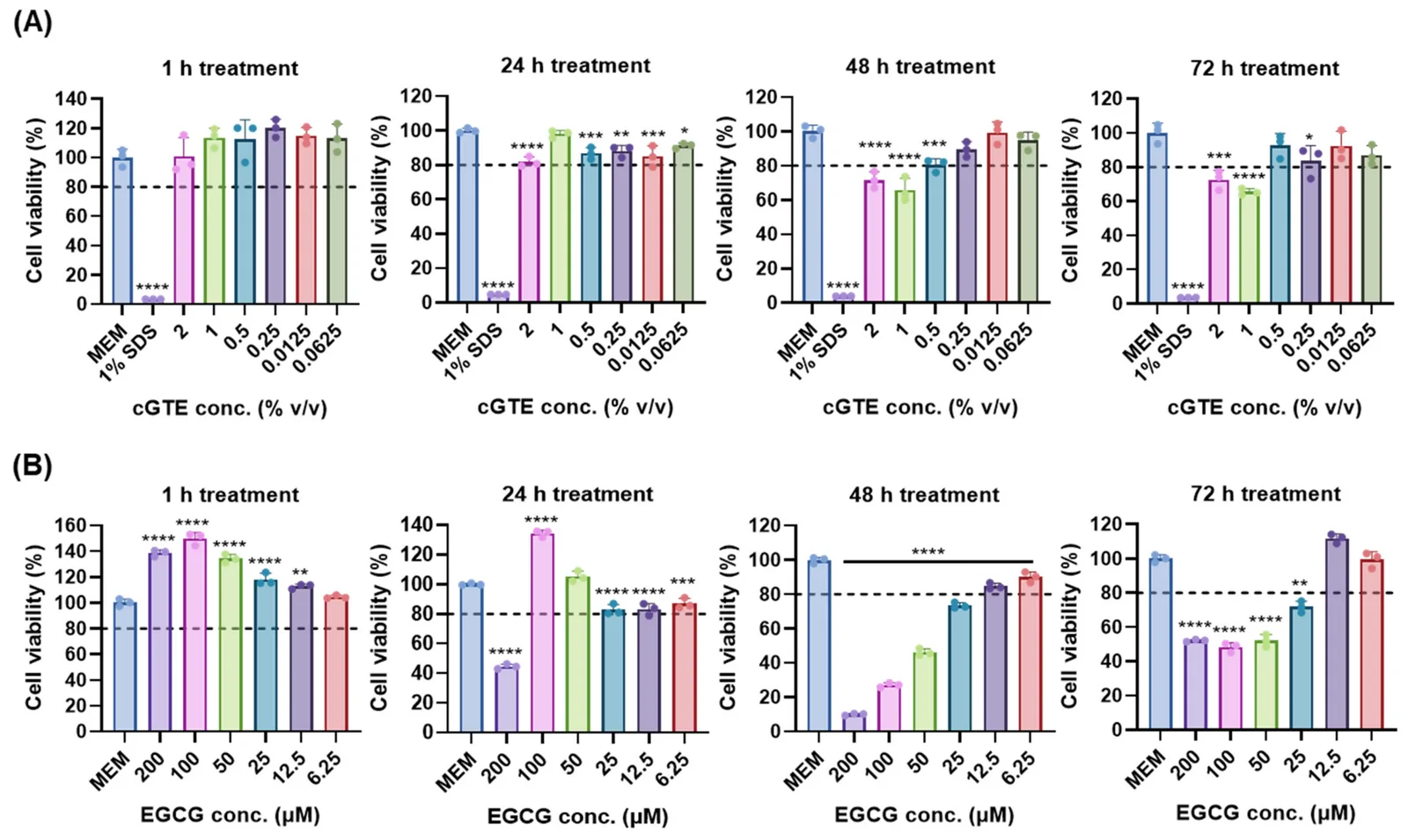
Cytotoxicity of the cGTE and EGCG determined by the MTT assay. (**A**) MDCK cells were treated with cGTE at concentrations of 0.0625–2% (*v*/*v*) for 1–72 h at 37 °C, and cell viability was determined using the MTT assay. (**B**) MDCK cells were treated with EGCG at concentrations of 6.25–200 μM for 1–72 h at 37 °C, followed by determination of cell viability using MTT assay. Minimum Essential Medium (MEM) and 1% sodium dodecyl sulfate (SDS) were used as negative and positive controls, respectively. Cell viability was expressed as a percentage relative to the MEM-treated control. The dashed horizontal line indicates 80% cell viability. Data are presented as mean ± SD (*n* = 3). Statistical significance was determined using one-way ANOVA followed by Dunnett’s post hoc test for multiple comparisons against the control group (MEM). Significant differences are indicated as * *p* < 0.05, ** *p* < 0.01, *** *p* < 0.001, and **** *p* < 0.0001 compared to the control. In the EGCG 48 h treatment, since all experimental groups shared the same statistical significance (****), they are collectively indicated by a single horizontal line to avoid visual crowding and save space.

The cytotoxicity of the cGTE preparation and EGCG was first evaluated in Madin-Darby canine kidney (MDCK) cells to determine suitable concentrations for subsequent antiviral assays. MDCK cells were treated with cGTE at concentrations of 0.0625–2% (*v*/*v*) or EGCG at concentrations of 6.25–200 μM for 1–72 h, and cell viability was subsequently measured by 3-(4,5-Dimethylthiazol-2-yl)-2,5-diphenyltetrazolium bromide (MTT) assay. cGTE showed relatively low cytotoxicity at concentrations of 0.5% or lower throughout the treatment period (Figure 1A). In contrast, treatment with higher concentrations of cGTE, particularly 1–2%, resulted in a reduction in cell viability after prolonged exposure, with the most pronounced decreases observed after 48 and 72 h. EGCG also showed a time- and concentration-dependent cytotoxicity profile, particularly at higher concentrations. Treatment with 200 μM EGCG markedly reduced cell viability after 24 h or longer, whereas 100 μM EGCG showed a substantial reduction in viability after 48 and 72 h (Figure 1B). In contrast, EGCG concentrations of 25 μM or lower generally maintained cell viability above approximately 80% during the tested period. Based on these results, non-cytotoxic concentrations were selected for subsequent antiviral experiments.

### 2.2. cGTE and EGCG Exhibit Direct Virucidal Activity Against Influenza A Viruses

**Figure 2:**
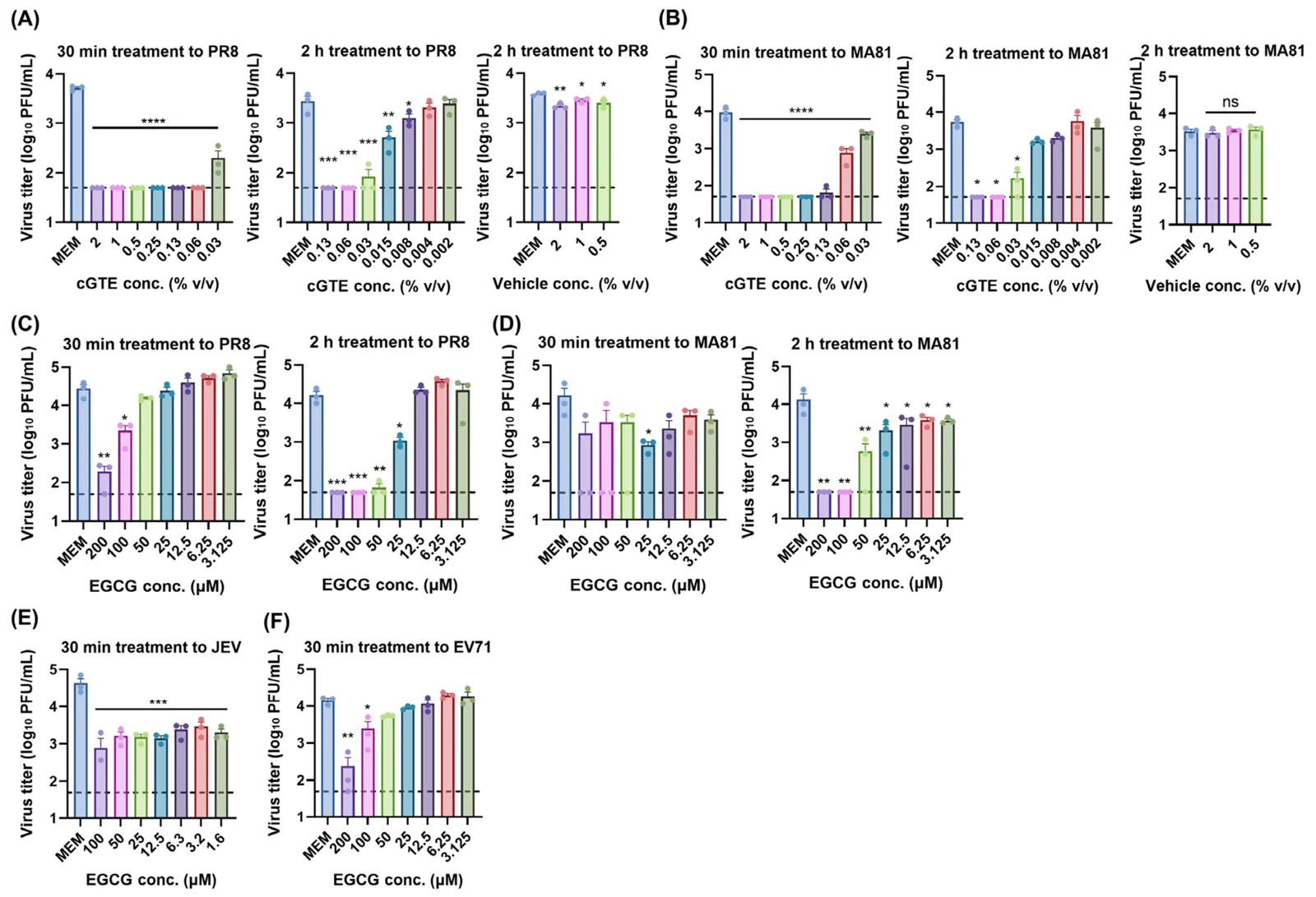
Virucidal activity of cGTE and EGCG against influenza A viruses, JEV, and EV71. 10^4^ plaque-forming units (PFU) of Influenza PR8 (**A**) or MA81 virus (**B**) were incubated with cGTE at the indicated concentrations for 30 min or 2 h at 37 °C. Vehicle control (0.5–2% *v*/*v*) was also included in the assay. 10^4^ PFU of PR8 (**C**) or MA81 virus (**D**) were incubated with EGCG at the indicated concentrations for 30 min or 2 h. 10^4^ PFU of JEV (**E**) or EV71 (**F**) was incubated with EGCG at the indicated concentrations for 30 min at 37 °C. Following treatment, the residual infectious virus was quantified by viral plaque assay. MEM-treated virus was used as the untreated control. The dashed horizontal line indicates the limit of detection of the plaque assay (1.699). Data are presented as mean ± SD (*n* = 3). Statistical significance was determined using one-way ANOVA followed by Dunnett’s post hoc test for multiple comparisons against the control group (MEM). Significant differences are indicated as * *p* < 0.05, ** *p* < 0.01, *** *p* < 0.001, and **** *p* < 0.0001 compared to the control; ‘ns’ indicates no statistical significance. A horizontal line spanning multiple groups indicates that all underlying groups achieve the same level of statistical significance.

To determine whether cGTE and EGCG directly inactivate influenza A viruses, viruses were exposed to cGTE or EGCG for defined periods, and the remaining infectious virus was quantified by viral plaque assay. cGTE exhibited a concentration-dependent virucidal effect against A/Puerto Rico/8/1934 (H1N1) (PR8) virus following 30 min of treatment, with viral titers reaching the detection limit at concentrations of 0.06% or higher (Figure 2A). Extending the treatment period to 2 h further enhanced the virucidal activity of cGTE, resulting in a detectable reduction in viral infectivity even at 0.008%. Treatment of PR8 virus with the vehicle control (0.5–2%, *v*/*v*) for 2 h resulted in an approximately 44% reduction in viral titers. However, this reduction was substantially lower than the virucidal effect observed with cGTE, suggesting that the observed virucidal activity was primarily attributable to the green tea extract rather than the other components of the vehicle. cGTE also effectively inactivated mouse-adapted influenza A/aquatic bird/Korea/w81/2005 (H5N2) (MA81) virus in a concentration- and time-dependent manner (Figure 2B). Treatment of MA81 virus with the vehicle control (0.5–2%) for 2 h resulted in an approximately 10% reduction in viral titers, indicating minimal virucidal activity of the vehicle components against MA81 virus. The virucidal activity of EGCG was subsequently examined against the same influenza A viruses. Against PR8 virus, EGCG produced a significant reduction in viral infectivity at concentrations of 100 μM or higher after 30 min of exposure, whereas a 2 h treatment resulted in significant viral inactivation at concentrations as low as 25 μM (Figure 2C). 30 min of EGCG treatment did not significantly reduce the infectious titer of MA81 virus, while extending the exposure period to 2 h resulted in a significant reduction at all tested concentrations (Figure 2D). To further investigate the spectrum of virucidal activity, EGCG was tested against Japanese encephalitis virus (JEV), an enveloped virus, and enterovirus A71 (EV71), a non-enveloped virus. EGCG exhibited concentration-dependent virucidal activity against both JEV and EV71 (Figure 2E,F). Collectively, these findings demonstrate that cGTE and EGCG directly inactivate influenza A viruses and that EGCG exhibits virucidal activity against both enveloped and non-enveloped viruses, suggesting a broader virucidal spectrum beyond influenza A viruses.

### 2.3. Effects of cGTE and EGCG on Different Stages of Influenza Virus Infection and Viral Surface Protein Functions

**Figure 3:**
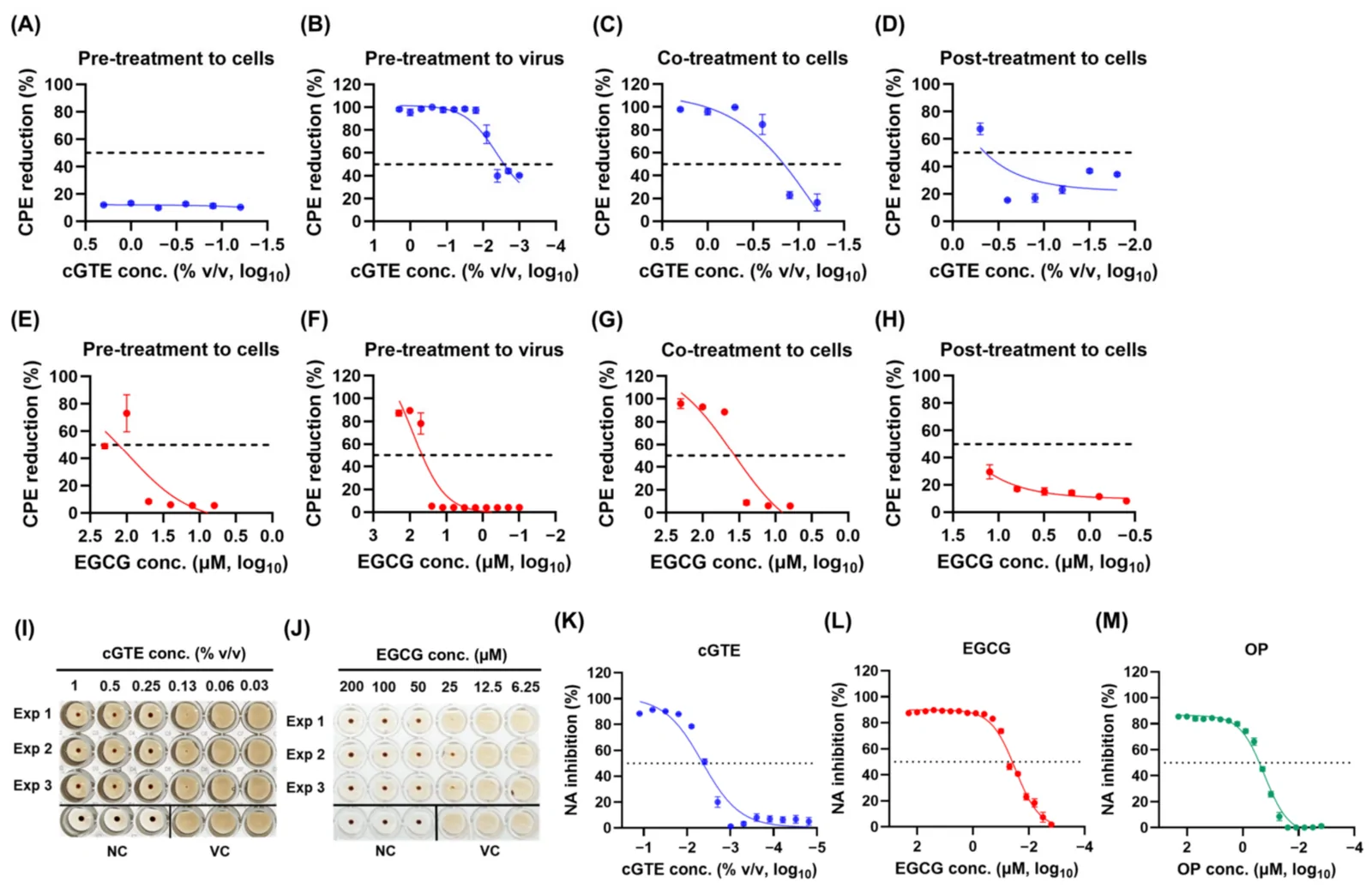
Antiviral activity of cGTE and EGCG assessed by TOA-CPE reduction experiments and their inhibitory effects on viral HA and NA. (**A**–**D**) Antiviral activity of cGTE against PR8 virus under four treatment conditions: pre-treatment to cells (**A**), pre-treatment to virus (**B**), co-treatment to cells (**C**), and post-treatment to cells (**D**). (**E**–**H**) Antiviral activity of EGCG under the corresponding treatment conditions: pre-treatment to cells (**E**), pre-treatment to virus (**F**), co-treatment to cells (**G**), and post-treatment to cells (**H**). For TOA experiments, MDCK cells were infected with 100 PFU of PR8 virus, and CPE was assessed at 72 h post-infection. CPE reduction was calculated relative to the virus-infected control and expressed as a percentage. The dashed horizontal line indicates 50% CPE reduction. (**I**,**J**) Representative images of the HI assay of PR8 virus pre-treated with the various concentrations of GTE (**I**) or EGCG (**J**). NC, negative control; VC, virus control. (**K**–**M**) Dose-dependent NI activity of cGTE (**K**), EGCG (**L**), and OP (**M**). The dashed horizontal line indicates 50% NA inhibition. IC_50_ values were determined by nonlinear regression using a four-parameter logistic dose–response model with a variable slope. The fitted model included the top and bottom plateaus as free parameters. IC_50_ values are reported with 95% confidence intervals (95% CIs).

To further characterize the stage of influenza virus infection affected by cGTE and EGCG, time-of-addition (TOA) experiments were performed, followed by a cytopathic effect (CPE) reduction assay. MDCK cells or PR8 virus were treated with cGTE or EGCG under four different conditions: pre-treatment to cells, pre-treatment to virus, co-treatment to cells, and post-treatment to cells. Both cGTE and EGCG exhibited different levels of antiviral activity depending on the timing of treatment. While cGTE showed little antiviral activity under the pre-treatment to cells condition (Figure 3A), it demonstrated dose-dependent antiviral activity under the other three modalities (Figure 3B–D). Among these, the most pronounced activity was observed under the pre-treatment to virus condition, yielding an IC_50_ value of 0.0026% (95% CI: 0.0018–0.0039%) (Figure 3B), followed by co-treatment to cells (IC_50_ = 0.19%; 95% CI: 0.16–0.23%) (Figure 3C) and post-treatment to cells (IC_50_ = 0.50%; 95% CI: 0.33–0.93%) (Figure 3D). These results suggest that cGTE exerts virucidal activity and may also interfere with viral attachment or entry. In contrast, EGCG exhibited a distinct antiviral profile. It showed potent antiviral activity primarily during the early stages of infection, specifically under pre-treatment to cells (IC_50_ = 54.9 µM; 95% CI: approximately 50–100 µM) (Figure 3E), pre-treatment to virus (IC_50_ = 40.05 µM; 95% CI: 35.91–49.26 µM) (Figure 3F), and co-treatment to cells, which demonstrated the highest potency with an IC_50_ of 36.88 µM (95% CI: 32.88–41.37 µM) (Figure 3G). However, EGCG displayed little to no inhibitory activity when added after viral entry into the cells (Figure 3H). Taken together, these results suggest that while both cGTE and EGCG possess potent virucidal activity and interfere with viral attachment or entry steps, they exhibit differential mechanisms during the post-entry phase of the viral life cycle. To further investigate whether cGTE and EGCG interfere with influenza virus surface protein functions, their effects on HA and NA activities were evaluated. Hemagglutination inhibition (HI) assay results showed that cGTE inhibited virus-mediated hemagglutination at concentrations of 0.25% or higher (Figure 3I), while EGCG also exhibited HI activity at 50 μM or higher (Figure 3J). These results indicate that both cGTE and EGCG can inhibit HA-mediated receptor binding, which is consistent with their CPE reduction activities under the co-treatment to cells condition. Both cGTE and EGCG also exhibited NA inhibition (NI) activity in a concentration-dependent manner, with IC_50_ values of 0.003679% (95% CI: 0.003410–0.003969) and 33.6 nM (95% CI: 28.6–38.6 nM), respectively (Figure 3K,L). As a positive control, oseltamivir phosphate (OP) showed potent NI activity, with an IC_50_ value of 173.5 nM (95% CI: 160.3–186.7 nM) (Figure 3M). These results demonstrate that both cGTE and EGCG inhibited the enzymatic activity of influenza virus NA in a concentration-dependent manner, with EGCG exhibiting greater inhibitory potency than OP under the conditions tested. Collectively, these findings indicate that the antiviral activities of cGTE and EGCG are associated not only with the direct inactivation of influenza virus particles but also with interference with the functions of the viral surface proteins HA and NA.

### 2.4. cGTE and EGCG Do Not Exhibit Bacterial Growth Inhibition in the Agar Well Diffusion Assay

**Figure 4:**
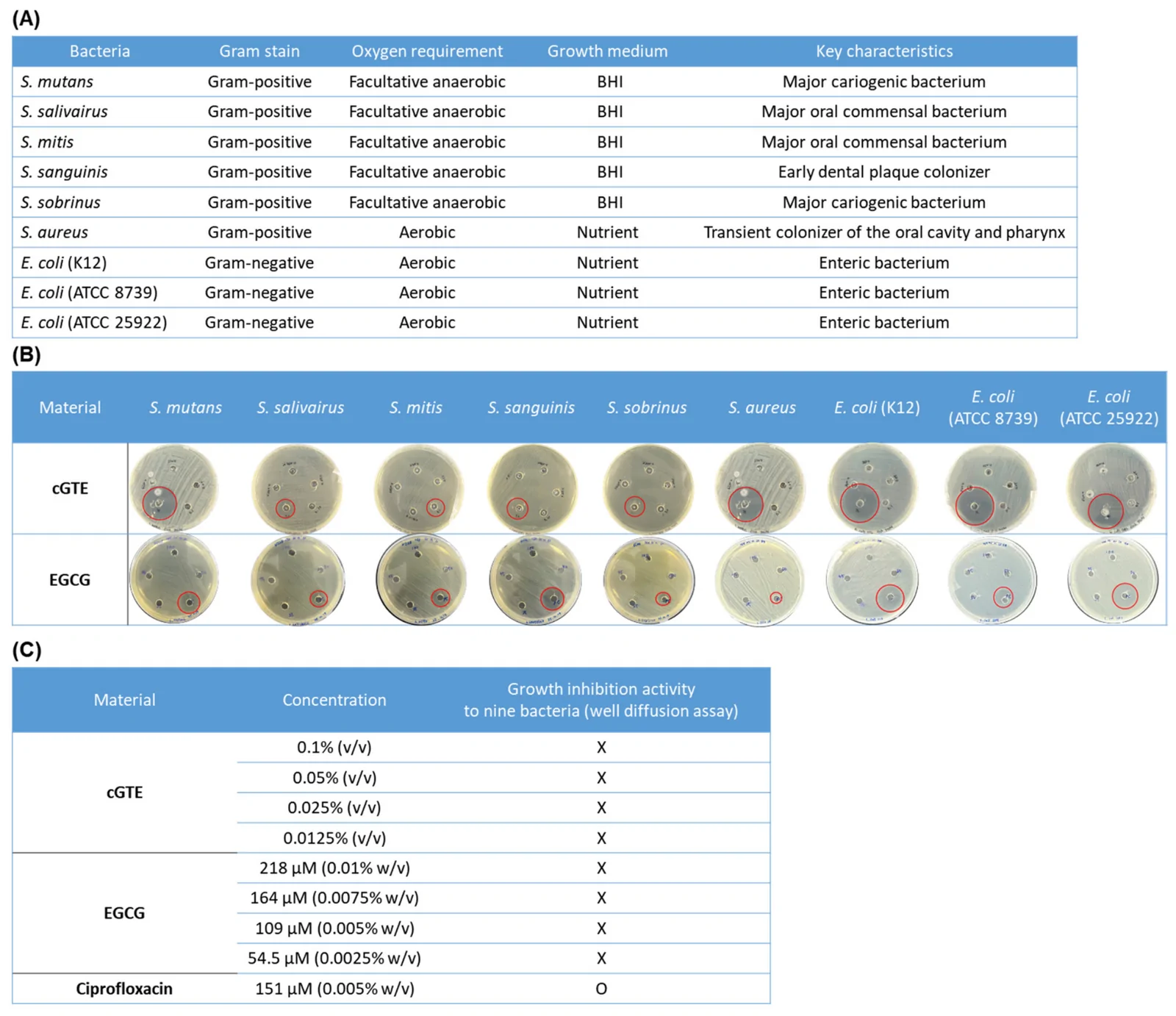
Antibacterial activities of cGTE and EGCG against oral bacteria and *E. coli* strains determined by the agar well diffusion assay. (**A**) Characteristics of the bacterial strains used in this study, including *S. mutans*, *S. salivarius*, *S. mitis*, *S. sanguinis*, *S. sobrinus*, *S. aureus*, *E. coli* K12, *E. coli* ATCC 8739, and *E. coli* ATCC 25922. (**B**) Representative images of the agar well diffusion assays showing the effects of cGTE and EGCG against the nine bacterial strains. cGTE was tested at concentrations of 0.1, 0.05, 0.025, and 0.0125% (*v*/*v*), whereas EGCG was tested at concentrations of 218, 164, 109, and 54.5 μM. CF (151 μM, 0.005%) was used as a positive control. Red circles indicate zones of inhibition produced by CF against the nine bacterial strains. Neither cGTE nor EGCG produced detectable zones of inhibition against any of the tested bacterial strains. (**C**) Summary of the agar well diffusion assay results for cGTE and EGCG at the indicated concentrations. The symbols ‘X’ and ‘O’ represent the absence and presence of growth inhibition activity, respectively.

Next, to evaluate the antibacterial activities of cGTE and EGCG, nine bacterial strains with distinct microbiological characteristics were selected for analysis. Five *Streptococcus* species, including *S. mutans*, *S. salivarius*, *S. mitis*, *S. sanguinis*, and *S. sobrinus*, were included as Gram-positive bacteria associated with the oral microbial environment. *S. aureus*, a Gram-positive facultative anaerobe that can transiently colonize the oral cavity and pharynx, was also included. In addition, three *E. coli* strains (K12, ATCC 8739, and ATCC 25922) were included as Gram-negative control strains. The bacterial species, Gram-staining characteristics, oxygen requirements, growth media, and major characteristics are summarized in Figure 4A. The antibacterial activities of cGTE and EGCG were initially evaluated using the agar well diffusion assay in which antimicrobial agents diffuse through the agar and inhibit bacterial growth, resulting in the formation of a clear zone of inhibition [23,24]. Ciprofloxacin (CF), a broad-spectrum antibacterial agent with demonstrated activity against both Gram-positive and Gram-negative bacteria, was included as a positive control [25]. CF exhibited clear antibacterial activity against all nine bacterial strains tested (Figure 4B). In contrast, no detectable inhibition zones were observed following treatment with cGTE or EGCG at any of the tested concentrations. These results are summarized in Figure 4C. In addition, our preliminary broth-based minimum inhibitory concentration (MIC) testing indicated that neither cGTE nor EGCG (from 0.0125% to 0.1%) produced measurable growth inhibition against *S. mutans* and *S. mitis*, suggesting that the MIC values for both materials were >0.1%. Thus, cGTE and EGCG showed no detectable growth inhibition under the conditions of either the agar well diffusion or MIC assays.

### 2.5. Direct Antibacterial Effects of cGTE and EGCG Against Oral Bacteria and E. coli Strains

**Figure 5:**
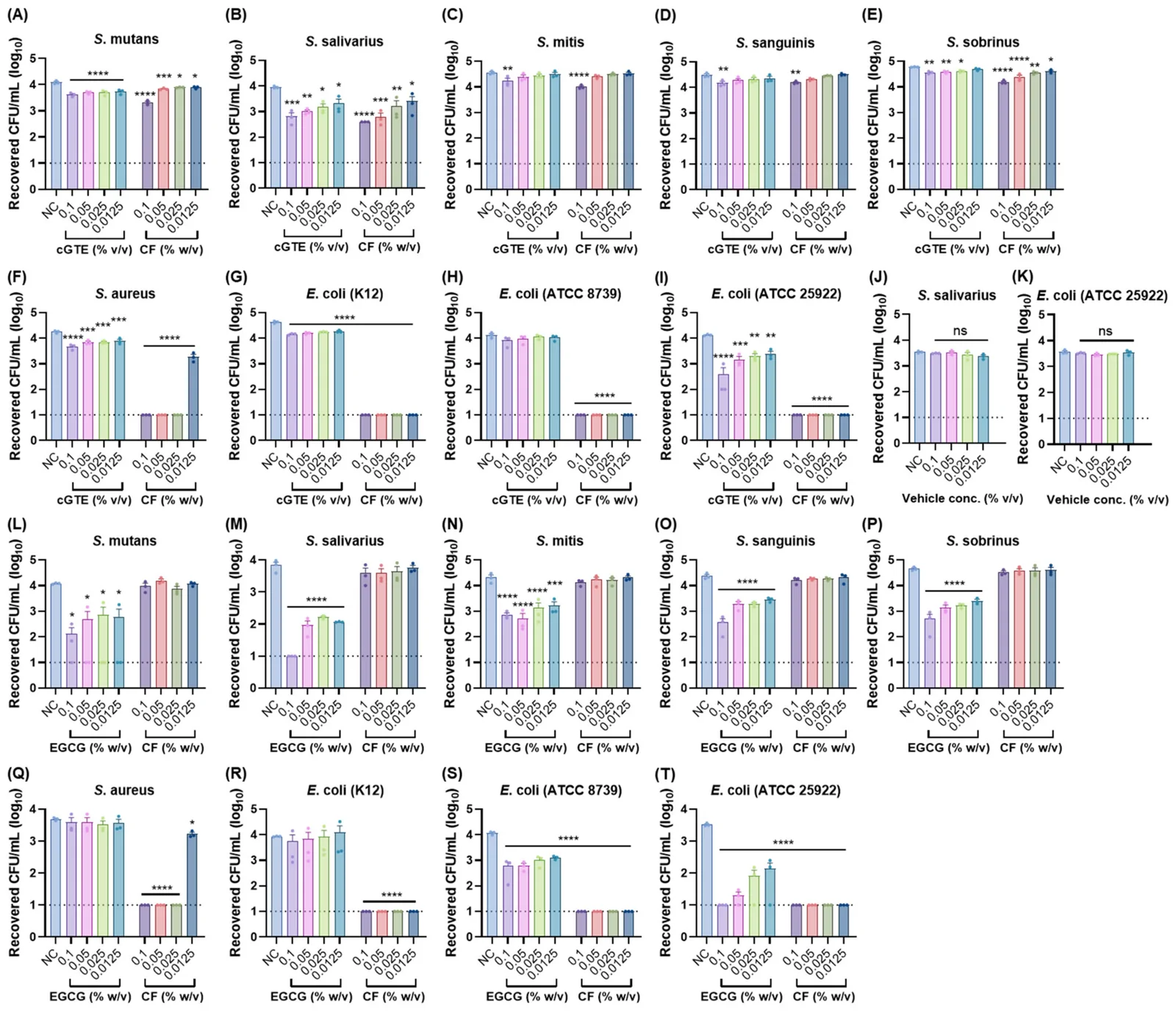
Direct antibacterial effects of cGTE and EGCG against oral bacteria and *E. coli strains.* Direct antibacterial activity of cGTE against *S. mutans* (**A**), *S. salivarius* (**B**), *S. mitis* (**C**), *S. sanguinis* (**D**), *S. sobrinus* (**E**), *S. aureus* (**F**), *E. coli* K12 (**G**), *E. coli* ATCC 8739 (**H**), and *E. coli* ATCC 25922 (**I**). Vehicle control was evaluated against *S. salivarius* (**J**) and *E. coli* ATCC 25922 (**K**). Bacterial suspensions (10,000 CFU) were treated with cGTE or vehicle control at concentrations of 0.0125–0.1% (*v*/*v*) for 1 h at 37 °C. Following treatment, viable bacteria were quantified by serial dilution and colony counting. CF was used as a control at the indicated concentrations. Direct antibacterial activities of EGCG against the same set of bacteria are shown (**L**–**T**). Asterisks indicate statistically significant differences compared with the NC. All concentrations of CF and EGCG are expressed as % (*w*/*v*). Data are presented as mean ± SD (*n* = 3). The dashed horizontal line in each graph indicates the assay limit of detection of 1.0 log_10_ CFU/mL (10 CFU/mL). Statistical significance was determined using one-way ANOVA followed by Dunnett’s post hoc test for multiple comparisons against the control group (NC). Significant differences are indicated as * *p* < 0.05, ** *p* < 0.01, *** *p* < 0.001, and **** *p* < 0.0001 compared to the control; ‘ns’ indicates no statistical significance. A horizontal line spanning multiple groups indicates that all underlying groups achieve the same level of statistical significance.

To quantitatively evaluate the direct antibacterial effects of cGTE and EGCG, each bacterial strain was exposed to the test materials for 1 h at 37 °C, after which the remaining viable bacteria were determined by colony counting. This assay was designed to assess the direct effects of cGTE and EGCG on bacterial viability under conditions analogous to the direct virucidal assay used for influenza viruses. CF was included as a positive control. The direct antibacterial activity of cGTE was evaluated against five oral Streptococcus species, *S. aureus*, and three *E. coli* strains (Figure 5A–I). cGTE significantly reduced the viable counts of *S. mutans*, *S. salivarius*, *S. mitis*, *S. sanguinis*, and *S. sobrinus* at one or more tested concentrations. cGTE also significantly reduced the viable counts of *S. aureus*. Among the *E. coli* strains, cGTE significantly reduced the viable counts of *E. coli* K12 and ATCC 25922, whereas no significant reduction was observed for *E. coli* ATCC 8739. The magnitude and statistical significance of the reduction varied depending on the bacterial species and cGTE concentration. No significant reduction in viable counts of *S. salivarius* or *E. coli* ATCC 25922 was observed following treatment with the vehicle control (0.0125–0.1%, v/v) (Figure 5J,K), suggesting that the antibacterial effects observed with cGTE were not attributable to the vehicle components under the conditions tested. EGCG was similarly evaluated under the same experimental conditions (Figure 5L–T). EGCG significantly reduced the viable counts of all five oral Streptococcus species, including *S. mutans*, *S. salivarius*, *S. mitis*, *S. sanguinis*, and *S. sobrinus*. Significant reductions were also observed for *E. coli* ATCC 8739 and ATCC 25922, whereas no significant reduction was observed for *E. coli* K12 or *S. aureus* under the conditions tested. The extent of EGCG-mediated reduction varied among the bacterial strains and concentrations. Overall, these results demonstrate that direct exposure to cGTE or EGCG for 1 h reduced bacterial viability in a strain-dependent manner, with both materials showing detectable antibacterial effects against the representative oral Streptococcus species, whereas their effects on *S. aureus* and *E. coli* varied among bacterial strains. These direct antibacterial effects were detected by quantitative colony counting despite the absence of detectable inhibition zones in the agar well diffusion assay.

### 2.6. Time-Dependent Direct Antibacterial Effects of cGTE and EGCG

**Figure 6:**
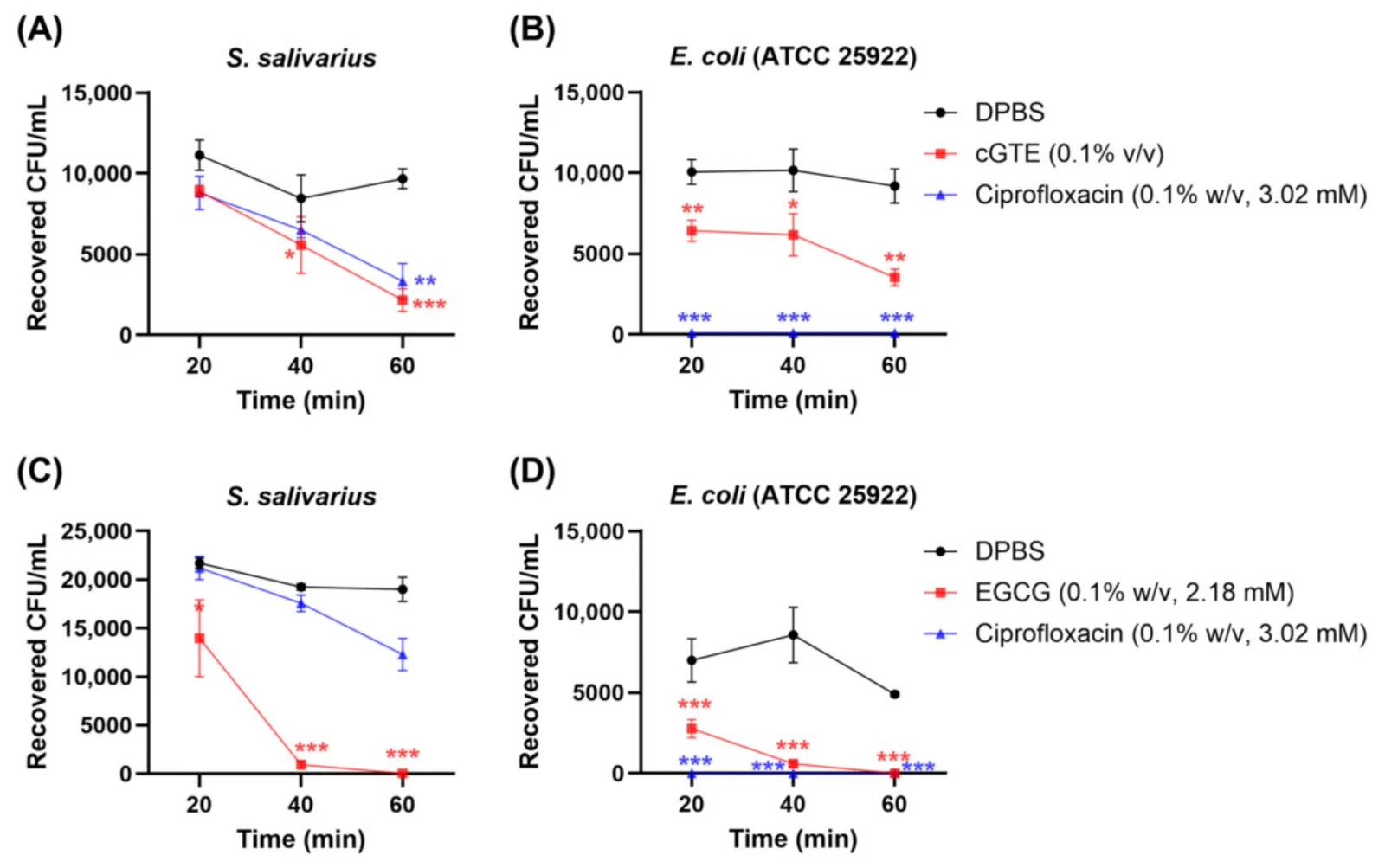
Time-dependent direct antibacterial activity of cGTE and EGCG. Time-dependent direct antibacterial activity of cGTE (0.1% *v*/*v*) against *S. salivarius* (**A**) and *E. coli* ATCC 25922 (**B**). Time-dependent antibacterial activity of EGCG (0.1% *w*/*v*) against *S. salivarius* (**C**) and *E. coli* ATCC 25922 (**D**). Bacterial suspensions (10,000 CFU) were incubated with cGTE or EGCG, for 20, 40, and 60 min, and viable bacterial counts were quantified by serial dilution and colony counting. DPBS was used as the untreated control, and CF was used as a positive control. The limit of detection for the bacterial count was 10 CFU/mL. Data are presented as mean ± SD (*n* = 3). Statistical analysis was performed using two-way ANOVA followed by Dunnett’s post hoc test for multiple comparisons against the control group (DPBS). Significant differences are indicated as * *p* < 0.05, ** *p* < 0.01, and *** *p* < 0.001 compared to the DPBS control at each corresponding time point.

To further evaluate the effects of exposure duration on bacterial viability, *S. salivarius* (Gram-positive) and *E. coli* ATCC 25922 (Gram-negative) were exposed to cGTE or EGCG for 20, 40, or 60 min, followed by quantification of the remaining viable bacteria by colony counting. CF was included as a positive control. Treatment with 0.1% cGTE reduced the viable counts of *S. salivarius* compared with DPBS, with significant reductions observed after 40 and 60 min of exposure (Figure 6A). For *E. coli* ATCC 25922, 0.1% cGTE significantly reduced viable bacterial counts at all tested exposure times, with the reduction becoming more pronounced after 60 min (Figure 6B). Similarly, 0.1% EGCG markedly reduced the viable counts of *S. salivarius*, with significant reductions observed after 40 and 60 min of exposure (Figure 6C). For *E. coli* ATCC 25922, 0.1% EGCG significantly reduced bacterial counts at all three exposure times, with viable bacteria reduced to near the detection limit after 60 min (Figure 6D). CF also reduced bacterial viability under the conditions tested, although the magnitude of reduction varied between the bacterial species and exposure times. Overall, both cGTE and EGCG reduced the viability of *S. salivarius* and *E. coli* ATCC 25922 following direct exposure, with greater reductions generally observed at longer exposure times. These findings support the direct antibacterial effects of cGTE and EGCG under the experimental conditions and demonstrate that the magnitude of bacterial reduction varied according to both bacterial strain and exposure duration.

## 3. Discussion

The present study demonstrates that cGTE and EGCG, a major catechin of green tea, possess dual antimicrobial activities against influenza A viruses and representative oral bacteria. A major strength of this study is the simultaneous evaluation of direct virucidal and antibacterial activities within a single experimental framework. Both cGTE and EGCG directly reduced influenza virus infectivity and bacterial viability, supporting their potential as multifunctional antimicrobial ingredients for oral and upper respiratory applications. Both materials exhibited concentration- and time-dependent virucidal activity against influenza A/H1N1 and A/H5N2 viruses, with particularly strong activity observed following direct treatment of the virus. The TOA-CPE reduction experiments further supported direct interaction with viral particles as an important component of their antiviral activity. In addition, both cGTE and EGCG demonstrated HI and NI activity, suggesting multi-target antiviral mechanisms.

Although the antiviral activity of green tea catechins, particularly EGCG, against influenza viruses has been reported previously, most studies have focused on individual catechins or specific stages of viral infection. In the present study, cGTE and EGCG were directly compared using complementary assays, including virucidal activity, TOA-CPE reduction, and HA- and NA-associated functions. Although EGCG reproduced several antiviral properties of cGTE, their antiviral effects showed distinct treatment-stage profiles, with differences observed in the extent of activity under the virus pre-treatment, co-treatment, and post-treatment conditions. These findings suggest that the antiviral activity of cGTE may not be attributable to EGCG alone and that other constituents may contribute to its overall activity. Thus, this study provides a comparative evaluation of a complex green tea extract and its major bioactive component within an application-oriented antiviral framework. A similar distinction was observed in their antibacterial profiles. Both cGTE and EGCG reduced the viability of the tested oral *Streptococcus* species, *S. aureus*, and *E. coli* strains following direct exposure; however, EGCG showed particularly strong antibacterial activity against the five oral *Streptococcus* species, with greater reductions than those observed with the ciprofloxacin control, whereas the activity of cGTE against these organisms was generally comparable to that of ciprofloxacin. In contrast, against *S. aureus* and the *E. coli* strains, both cGTE and EGCG showed significant but generally weaker reductions than ciprofloxacin. These findings indicate that although cGTE and EGCG share broad antibacterial activity, the magnitude of their activity differs depending on the bacterial species and may reflect differences in the active constituents and their interactions with bacterial cells.

A notable finding was that both cGTE and EGCG exhibited antibacterial activity against Gram-positive and Gram-negative bacteria despite producing no detectable inhibition zones in the agar well diffusion assay. In contrast, CF produced clear inhibition zones and reduced bacterial viability following direct exposure. This discrepancy may be related to differences in the physicochemical properties and antimicrobial mechanisms of the tested materials. Agar diffusion depends not only on antimicrobial potency but also on solubility and diffusion through the agar matrix; therefore, the absence of an inhibition zone does not necessarily indicate a lack of antibacterial activity, particularly for plant-derived polyphenols such as EGCG [26]. Previous studies have similarly reported that EGCG can exhibit bactericidal activity under direct exposure conditions, while its activity in diffusion-based assays may be influenced by its diffusion characteristics and the concentration applied [18,27]. In the direct antibacterial assay used in the present study, bacteria were exposed directly to cGTE or EGCG, allowing the test materials to interact with bacterial cells without the additional diffusion step required in the agar assay. Thus, differences in exposure conditions between the two assays may contribute to the observed discrepancy in antibacterial activity. Although EGCG may act through interactions with bacterial surface- or membrane-associated components, the underlying mechanisms were not directly examined in the present study and warrant further investigation.

Importantly, the simultaneous virucidal and antibacterial activities observed in this study support the potential application of cGTE and EGCG in locally applied antimicrobial formulations. Previous studies have investigated the potential of tea-based interventions, including green tea gargling and catechin consumption, for reducing the risk of influenza and other upper respiratory tract infections [28,29]. Given that both influenza virus infectivity and bacterial viability were reduced following direct exposure in the present study, cGTE and EGCG may be promising candidates for further development as spray-type formulations intended for the oral cavity or nasal passages. The concept of locally applied natural antimicrobial formulations has also been explored in previous studies. Herbal oral spray formulations have been reported to exhibit both antiviral and antibacterial activities against pathogens relevant to the oral and upper respiratory environments, supporting the feasibility of multifunctional antimicrobial products [30,31]. In this context, the present findings provide a mechanistic basis for the potential application of cGTE and EGCG in locally applied formulations by demonstrating direct reductions in both influenza virus infectivity and bacterial viability. Such formulations could provide direct contact with respiratory viruses and oral microorganisms and potentially reduce microbial infectivity or viability at the site of exposure. Thus, the dual antimicrobial properties of cGTE and EGCG may provide an advantage over formulations targeting only a single type of microorganism.

This study has several limitations. Although direct antibacterial activity was demonstrated, the underlying mechanisms responsible for bacterial killing were not investigated. Future studies should therefore examine the effects of cGTE and EGCG on bacterial membrane integrity, permeability, oxidative stress, and other cellular targets, as multiple mechanisms have been proposed for the antibacterial activity of green tea catechins [32,33,34]. Furthermore, it is important to acknowledge that potential carry-over effects of cGTE and EGCG could not be completely excluded in our experimental design. Although serial dilution and washing steps were employed, residual compounds might have exerted continued antimicrobial activity during the subsequent viral adsorption or bacterial colony formation periods. Therefore, the observed reductions should be interpreted cautiously as a decrease in recoverable infectious viral titers and viable bacterial counts under these specific assay conditions, rather than definitive irreversible inactivation. Another limitation is the lack of detailed chemical standardization for the commercial cGTE formulation. Although we obtained its basic composition (1.25% green tea extract, 30% butylene glycol, and 3% 1,2-hexanediol), specific information regarding individual catechin content, including EGCG levels, was unavailable from the manufacturer due to proprietary constraints. Consequently, we could not determine the exact contribution of specific chemical components within cGTE to the observed antimicrobial effects, and further standardization will be required in future translational studies. In addition, because the present findings were obtained in vitro, further studies using physiologically relevant oral and nasal models will be required to evaluate formulation stability, mucosal safety, retention time, and antimicrobial activity under conditions more closely resembling actual use. In particular, studies using repeated topical administration should evaluate the mucosal retention and local exposure of cGTE and EGCG, as repeated administration may result in increased local concentrations or accumulation depending on the formulation and dosing frequency, potentially affecting mucosal safety. Although the present in vitro study did not assess the residence time or half-life of cGTE and EGCG on the oral or nasal mucosal surface, these pharmacokinetic parameters may vary substantially depending on the formulation, mucosal environment, and route of administration. Such studies would help determine whether the relatively low cytotoxicity observed at lower concentrations in vitro is maintained under repeated-use conditions.

In conclusion, cGTE and EGCG exhibited dual direct antimicrobial activities against influenza viruses and representative oral bacteria. The simultaneous demonstration of virucidal and antibacterial activities is a key strength of this study, supporting the potential of these green tea-derived materials as multifunctional natural antimicrobial ingredients. These findings provide a basis for the development of oral or nasal spray formulations designed to reduce microbial infectivity and viability through direct local exposure, although further mechanistic and formulation studies are required to establish their practical effectiveness and safety.

## 4. Materials and Methods

### 4.1. Reagents, Animal Cell Lines, and Viruses

The cGTE was obtained from Dermalab (Wonju, Republic of Korea). According to the manufacturer, the commercial formulation contained 1.25% (*w*/*v*) green tea leaf extract, with butylene glycol (30%, *v*/*v*) and 1,2-hexanediol (3%, *v*/*v*) as the principal liquid formulation components, dissolved in an aqueous vehicle. The undiluted commercial formulation was used as the cGTE stock solution, and all experimental concentrations were prepared by volumetric dilution of the stock solution with the appropriate medium or buffer. Thus, a cGTE concentration of 0.1% (*v*/*v*) corresponded to a 1:1000 dilution of the undiluted commercial formulation. All working solutions were prepared immediately before each experiment. EGCG was sourced from Hunan Sunshine Bio-tech (Changsha, Hunan, China), whereas ciprofloxacin and OP were supplied by Sigma-Aldrich (St. Louis, MO, USA). The concentrations of these compounds were expressed as molar concentrations (M) or as weight/volume percentages (*w*/*v*), as appropriate. MDCK, Vero, and BHK-21 cells were obtained from the American Type Culture Collection (Manassas, VA, USA). MDCK cells were propagated in MEM (Gibco, Waltham, MA, USA), and Vero and BHK-21 cells in DMEM (Gibco). Both media were supplemented with 10% FBS (HyClone, Logan, UT, USA) and ZellShield^®^ antibiotic (Minerva Biolabs GmbH, Berlin, Germany). Cell cultures were incubated at 37 °C under a humidified atmosphere containing 5% CO_2_. The viral panel comprised influenza PR8 and MA81, EV71, and JEV (genotype III, Nakayama strain).

### 4.2. In Vitro Cytotoxicity Assay

The cytotoxic effects of the cGTE and EGCG on MDCK cells were assessed using the MTT assay (Sigma-Aldrich). Briefly, cells were seeded in 96-well plates and treated with various concentrations of cGTE or EGCG for different incubation periods (1–72 h) at 37 °C. Following treatment, 10 μL of MTT solution was added to each well, and the plates were incubated for 3 h at 37 °C. Subsequently, 100 μL of DMSO was added to dissolve the resulting formazan crystals. The plates were further incubated at room temperature for 30 min, and absorbance was measured at 570 nm using a microplate reader.

### 4.3. Viral Plaque Assay and Virucidal Assay

Infectious virus titers were quantified by plaque formation using MDCK cells for influenza virus, Vero cells for EV71, and BHK-21 cells for JEV. Viral titers were expressed as PFU. Briefly, virus preparations were serially diluted 10-fold in the appropriate culture medium, and 200 μL of each of the 1:10, 1:100, and 1:1000 dilutions were inoculated onto confluent cell monolayers in 12-well plates. Viral titers were calculated based on the number of plaques and the corresponding dilution factor. Under these assay conditions, the limit of detection was 1.699 log_10_ PFU/mL (50 PFU/mL). After adsorption for 1–2 h at 37 °C, the inoculum was removed, and the cells were gently washed with PBS. The monolayers were then covered with DMEM containing 1% low-melting-point agarose (Lonza, Walkersville, MD, USA) and antibiotic-antimycotic solution (GenDEPOT, Hanam, Republic of Korea). Following incubation at 37 °C in a humidified atmosphere containing 5% CO_2_ for 2–3 days, plaques were visualized by fixing the cells with 4% paraformaldehyde and carefully removing the agarose overlay. The fixed monolayers were subsequently stained with 1% crystal violet solution, and the number of plaques was counted to determine infectious virus titers. For the virucidal assay, 10^4^ PFU of each virus was mixed with various concentrations of cGTE or EGCG and maintained at 37 °C for 30 min or 2 h. After treatment, the mixtures were serially diluted and subjected to plaque assay to determine the remaining infectious virus titers.

### 4.4. TOA-CPE Experiments

TOA experiments were conducted to determine the stage of the influenza virus replication cycle affected by cGTE or EGCG. Antiviral activity was examined under four treatment conditions according to the timing of material exposure relative to viral infection: pre-treatment to cells, pre-treatment to virus, co-treatment to cells, and post-treatment to cells. MDCK cells seeded in 96-well plates were infected with 100 PFU of influenza PR8 virus, and cell viability was assessed by the MTT assay at 72 h post-infection. Antiviral efficacy was determined based on the reduction in virus-induced CPE compared with the untreated virus control. Pre-treatment to cells. To assess whether prior exposure of host cells to cGTE or EGCG could protect against subsequent viral infection, MDCK cells were treated with serially diluted test materials for 1 h at 37 °C. The cells were then washed with PBS to remove residual compounds and subsequently inoculated with 100 PFU of influenza virus. Following a 1 h adsorption period, the inoculum was removed, the cells were washed again with PBS, and fresh culture medium was added. Pre-treatment to virus. To examine the direct virucidal effect of cGTE or EGCG on viral particles, two-fold serial dilutions of each material were mixed with approximately 100 PFU of influenza virus and incubated at 37 °C for 2 h. The resulting mixtures were then transferred onto MDCK cell monolayers and allowed to adsorb for 1 h at room temperature. The inoculum was subsequently removed, and the cells were washed to eliminate unbound virus and residual test materials before the addition of fresh culture medium. Co-treatment to cells. To determine whether cGTE or EGCG interferes with the early phase of viral infection, serially diluted materials were combined with influenza virus immediately before inoculation onto MDCK cell monolayers. After a 1 h adsorption period, the inoculum was removed, and the cells were washed with PBS to eliminate unbound virus and residual materials. Fresh culture medium was then added for the remainder of the incubation period. Post-treatment to cells. To investigate the effects of cGTE or EGCG on viral replication following viral entry, MDCK cells were first inoculated with influenza virus and allowed to undergo adsorption for 1 h. The inoculum was then removed, and the cells were washed with PBS. Fresh culture medium containing the indicated concentrations of the test materials was subsequently added and maintained throughout the remaining incubation period.

### 4.5. HI Assay

Hemagglutination activity was first quantified by determining the hemagglutination units (HAU) of the influenza virus stocks. Briefly, two-fold serial dilutions of the virus preparations were made in PBS, and 50 μL of each dilution was transferred to V-bottom 96-well plates. An equal volume of 1% chicken red blood cells (cRBCs) suspension (Innovative Research, Novi, MI, USA) was subsequently added, and the plates were incubated at 4 °C for 1 h. The HAU titer was defined as the reciprocal of the highest virus dilution that produced complete hemagglutination, characterized by the formation of a diffuse erythrocyte lattice rather than a compact cell pellet. For the HI assay, cGTE or EGCG was combined with 4 HAU of influenza virus and preincubated at 37 °C for 30 min in V-bottom microplates. Following treatment, 50 μL of 1% cRBCs suspension was added to the reaction mixtures, which were subsequently maintained at 4 °C for 1 h. HI activity was assessed visually based on the erythrocyte pattern formed in each well, with inhibition indicated by the formation of a distinct erythrocyte pellet.

### 4.6. NI Assay

NA activity of influenza virus was first determined using an enzyme-linked lectin assay. Briefly, 96-well immunoplates were coated with 100 μL of fetuin (Sigma-Aldrich) per well and kept at 4 °C for 18 h. The coated plates were then washed, and two-fold serial dilutions of influenza virus were applied to the wells. Following incubation at 37 °C for 1 h, the plates were washed and treated with 100 μL of lectin (Sigma-Aldrich) per well. After a further 1 h incubation at room temperature, the wells were washed again, followed by the addition of 100 μL of tetramethylbenzidine (TMB) substrate (Thermo Scientific, Waltham, MA, USA). Color development was allowed to proceed for 5 min and was terminated by adding 50 μL of 2 N H_2_SO_4_ to each well. Absorbance was subsequently recorded at 450 nm. For the NI assay, two-fold serial dilutions of cGTE, EGCG, or OP were prepared and mixed with influenza virus standardized to an NA activity corresponding to an OD_450_ value of 1. The virus–material mixtures were maintained at 37 °C for 30 min and subsequently transferred to fetuin-coated plates. Residual NA activity was determined using the same procedure described above.

### 4.7. Bacterial Strains and Culture Conditions

Five oral Streptococcus species, including *S. mutans* (KCTC 3065), *S. salivarius* (KCTC 3960), *S. mitis* (KCTC 13047), *S. sanguinis* (KCTC 3284), and *S. sobrinus* (KCTC 3288), together with *S. aureus* (KCTC 1621) and three *E. coli* strains K12 (KCTC 1116), ATCC 8739 (KCTC 2571), and ATCC 25922 (KCTC 1682), were obtained from the Korean Collection for Type Cultures (KCTC, Jeongeup, Republic of Korea). Lyophilized bacterial preparations were reconstituted with sterile distilled water and subsequently streaked onto the appropriate agar media using sterile inoculating loops. *S. mutans*, *S. salivarius*, *S. mitis*, *S. sanguinis*, and *S. sobrinus* were cultured on BHI agar (KisanBio, Seoul, Republic of Korea) whereas *S. aureus* and all three *E. coli* strains were grown on Nutrient agar (KisanBio). Following inoculation, the plates were sealed with Parafilm and incubated at 37 °C for 24 h under conventional atmospheric conditions without controlled CO_2_ or anaerobic incubation. An isolated colony from each strain was then transferred to the corresponding broth medium and cultivated at 37 °C with agitation at 180 rpm until the optical density at 660 nm reached 0.5–0.9. For long-term preservation, the resulting bacterial cultures were combined with 50% (*v*/*v*) glycerol and the respective broth medium at a volume ratio of 1:1:1. This procedure yielded bacterial stocks containing approximately 16.7% (*v*/*v*) glycerol, which were stored at −80 °C until subsequent experiments.

### 4.8. Agar Well Diffusion Assay

The antibacterial activities of cGTE and EGCG were assessed by the agar well diffusion method. Briefly, bacterial suspensions prepared from freshly grown cultures were adjusted to a 0.5 McFarland standard, corresponding to approximately 1.5 × 10^8^ CFU/mL in DPBS. The standardized suspensions were uniformly inoculated onto the surface of the appropriate agar media using sterile cotton swabs. Five wells were then created in each agar plate using a sterile pipette tip. Aliquots of 20 μL of the respective test materials were dispensed into the wells and allowed to diffuse into the agar. After sufficient diffusion into the agar, the plates were sealed with Parafilm and incubated at 37 °C for 24 h. Following incubation, antibacterial activity was determined by measuring the diameter of the clear zones of growth inhibition surrounding each well.

### 4.9. Minimum Inhibitory Concentration (MIC) Assay

The MICs of cGTE and EGCG were determined by broth microdilution against *S. mutans* and *S. mitis* strains. The test materials were serially two-fold diluted in BHI broth to achieve a concentration range of 0.0125% to 0.1% in a total volume of 100 μL per well in 96-well plates. Logarithmic-phase bacterial inocula were added to each well at a final density of 5 × 10^5^ CFU/mL. Wells containing BHI broth inoculated with bacteria without test materials served as positive controls (growth controls), while wells with BHI broth only served as negative controls (sterility controls). After 24 h incubation at 37 °C under conventional atmospheric conditions, bacterial growth was assessed visually by turbidity and quantified at OD_660_. The MIC was defined as the lowest concentration inhibiting visible bacterial growth. All assays were performed in triplicate.

### 4.10. Direct Antibacterial Activity Assay

The direct antibacterial activities of cGTE and EGCG were evaluated by determining the survival of bacteria following treatment with the test materials. Bacterial suspensions containing approximately 10^4^ CFU were prepared in DPBS and exposed to cGTE or EGCG at final concentrations of 0.1%, 0.05%, 0.025%, or 0.0125% in a total volume of 1 mL. CF was included as a positive control at the corresponding concentrations. The bacterial suspensions were subsequently maintained at 37 °C for 1 h. After treatment, the resulting suspensions were plated without further removal of the test materials. For determination of recoverable CFUs, the original suspensions, 1:10, and 1:100 dilutions were plated, as appropriate. A 100 μL aliquot of each suspension or dilution was spread onto Nutrient agar plates for *S. aureus* and three *E. coli* strains, whereas BHI agar was used for the remaining oral bacterial strains. Under these experimental conditions, the limit of detection for the bacterial recovery assay was 10 CFU/mL (1.0 log_10_ CFU/mL). Sterile spreaders were prepared by soaking them in 100% ethanol, followed by flame sterilization and cooling before use. The inoculated plates were incubated at 37 °C for 24 h. Bacterial viability was quantified by enumerating the colonies formed on the agar plates, and survival was expressed based on the number of viable CFUs recovered after treatment.

### 4.11. Time-Kill Kinetics

The time-dependent direct antibacterial effects of cGTE and EGCG were investigated using *S. salivarius* and *E. coli* ATCC 25922, which showed high susceptibility among the tested Gram-positive and Gram-negative bacteria, respectively. Bacterial suspensions were prepared in DPBS at a final volume of 1 mL, containing 10^4^ CFU bacteria and 0.1% cGTE or EGCG. CF was included as a positive control. All bacterial suspensions were maintained at 37 °C, and samples were collected after 20, 40, and 60 min of treatment. After treatment, the resulting suspensions were plated without further removal of the test materials. For determination of recoverable CFUs, a 100 μL aliquot of the original suspensions, 1:10, and 1:100 dilutions were spread onto the appropriate agar medium. The plates were incubated at 37 °C for 24 h and bacterial survival at each time point was determined by enumerating the viable colonies recovered from the agar plates. The limit of detection for the bacterial recovery assay was 10 CFU/mL (1.0 log_10_ CFU/mL).

### 4.12. Statistical Analysis

All experiments were independently performed at least three times, and the data are presented as the mean ± standard deviation (SD). Statistical analyses were conducted using GraphPad Prism version 9.0. Comparisons between more than two groups were performed using one-way analysis of variance (ANOVA) or two-way ANOVA followed by Dunnett’s multiple-comparisons test, as appropriate. Statistical significance is indicated as follows: *p* < 0.05 (*), *p* < 0.01 (**), *p* < 0.001 (***), and *p* < 0.0001 (****). IC_50_ values were determined by nonlinear regression using a four-parameter logistic dose–response model with a variable slope. The fitted model included the top and bottom plateaus as free parameters. IC_50_ values are reported with 95% confidence intervals.

## Data Availability

The original contributions presented in this study are included in the article. Further inquiries can be directed to the corresponding author.

## Abbreviations

The following abbreviations are used in this manuscript:

cGTE	Commercial green tea extract
EGCG	Epigallocatechin gallate
PR8	A/Puerto Rico/8/1934 (H1N1)
MA81	Mouse-adapted influenza A/aquatic bird/Korea/w81/2005 (H5N2)
HA	Hemagglutinin
NA	Neuraminidase
MDCK	Madin-Darby canine kidney
EV71	Enterovirus A71
TOA	Time-of-addition
CPE	Cytopathic effect
HI	Hemagglutination inhibition
NI	Neuraminidase inhibition
CF	Ciprofloxacin
MTT	3-(4,5-Dimethylthiazol-2-yl)-2,5-diphenyltetrazolium bromide
JEV	Japanese encephalitis virus
PFU	Plaque-forming unit
MOI	Multiplicity of infection
HAU	Hemagglutination unit
cRBC	Chicken red blood cell
OP	Oseltamivir phosphate
MEM	Minimum Essential Medium
DMEM	Dulbecco’s Modified Eagle’s Medium
DPBS	Dulbecco’s phosphate-buffered saline
FBS	Fetal bovine serum
BHI	Brain heart infusion
SDS	Sodium dodecyl sulfate
MIC	Minimum inhibitory concentration
